# Cone Snails: A Big Store of Conotoxins for Novel Drug Discovery

**DOI:** 10.3390/toxins9120397

**Published:** 2017-12-07

**Authors:** Bingmiao Gao, Chao Peng, Jiaan Yang, Yunhai Yi, Junqing Zhang, Qiong Shi

**Affiliations:** 1 Hainan Provincial Key Laboratory of Research and Development of Tropical Medicinal Plants, Hainan Medical University, Haikou 571199, China; gaobingmiao1982@163.com or hy0207083@hainmc.edu.cn; 2 Shenzhen Key Lab of Marine Genomics, Guangdong Provincial Key Lab of Molecular Breeding in Marine Economic Animals, BGI Academy of Marine Sciences, BGI Marine, BGI, Shenzhen 518083, China; pengchao@genomics.cn (C.P.); yiyunhai@genomics.cn (Y.Y.); 3 Micro Pharmtech, Ltd., Wuhan 430075, China; jyang@micropht.com; 4 BGI Education Center, University of Chinese Academy of Sciences, Shenzhen 518083, China

**Keywords:** conotoxin, cone snail, transcriptome, proteome, drug development

## Abstract

Marine drugs have developed rapidly in recent decades. Cone snails, a group of more than 700 species, have always been one of the focuses for new drug discovery. These venomous snails capture prey using a diverse array of unique bioactive neurotoxins, usually named as conotoxins or conopeptides. These conotoxins have proven to be valuable pharmacological probes and potential drugs due to their high specificity and affinity to ion channels, receptors, and transporters in the nervous systems of target prey and humans. Several research groups, including ours, have examined the venom gland of cone snails using a combination of transcriptomic and proteomic sequencing, and revealed the existence of hundreds of conotoxin transcripts and thousands of conopeptides in each *Conus* species. Over 2000 nucleotide and 8000 peptide sequences of conotoxins have been published, and the number is still increasing quickly. However, more than 98% of these sequences still lack 3D structural and functional information. With the rapid development of genomics and bioinformatics in recent years, functional predictions and investigations on conotoxins are making great progress in promoting the discovery of novel drugs. For example, ω-MVIIA was approved by the U.S. Food and Drug Administration in 2004 to treat chronic pain, and nine more conotoxins are at various stages of preclinical or clinical evaluation. In short, the genus *Conus*, the big family of cone snails, has become an important genetic resource for conotoxin identification and drug development.

## 1. Introduction

Forming the biggest single genera of living marine invertebrates [1], cone snails are composed of various carnivorous predators. They are usually classified into three groups depending on their feeding habits: worm hunters (vermivorous), mollusk hunters (molluscivorous), and fish hunters (piscivorous) [1,2,3]. There are now around 700 *Conus* species, with the majority distributed throughout tropical and subtropical waters, such as the South China Sea, Australia, and the Pacific Ocean [4].

The venom gland of cone snails can secrete large amounts of unique neurotoxic peptides, commonly referred to as conopeptides or conotoxins, and most conotoxins are rich in disulfide bridges with many pharmacological activities [5,6]. Each *Conus* species typically possesses an average of 100–200 conotoxins as potential pharmacological targets [7]. More than 80,000 natural conotoxins have been estimated to exist in various cone snails around the world [7,8,9]. Therefore, the cone snails construct the largest library of natural drug candidates for the development of marine drugs.

Usually, conotoxins are categorized into many different families based on the types of their molecular targets and corresponding pharmacological activities [9,10]. Their structures and functions are highly diverse and mainly target membrane proteins, particularly ion channels, membrane receptors, and transporters. Some toxins that have specific targets and short sequences and are easy to synthesize have been developed as drug leads and effective research agents for distinguishing subtypes of molecular targets [10,11]. The most well-known commercial conotoxin is ω-MVIIA (ziconotide), which has been derived from the venom of a fish-hunting *C. magus* species, and approved by the U.S. Food and Drug Administration (FDA) to treat chronic pain in serious cancer and AIDS patients [12,13]. Conotoxins are now increasingly undergoing development for the treatment of multiple diseases including pain, Alzheimer’s disease, Parkinson’s disease, cardiac infarction, hypertension, and various neurological diseases [14,15,16]. Therefore, conotoxins, being important potential therapeutic targets due to the physiological roles that they play, are a practical prospect with wide implications in the neuroscience research field.

## 2. Diversity of Cone Snails

### 2.1. Various Phenotypes

**Figure 1:**
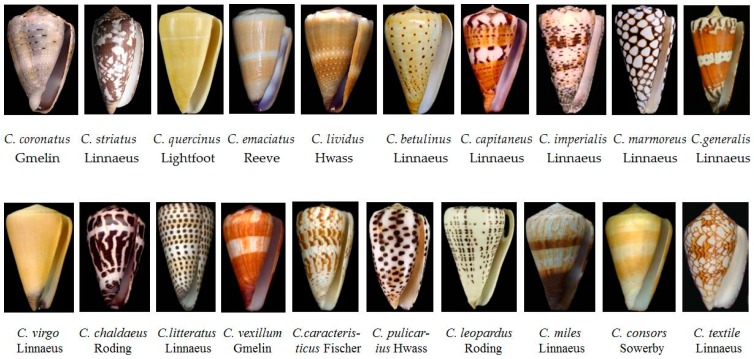
Twenty most abundant *Conus* species in the South China Sea.

Cone snails have been of interest as collector’s items for a long time due to their beautiful patterned shells (Figure 1), and the identification of *Conus* species is mainly based on their morphology and color of shell. The genus *Conus* is a member of the most diverse and taxonomically complex superfamily, Conoidea. According to recent estimations, it includes around 700 species, and most of them still remain undescribed [17]. However, species determination of live cone snails using shell characteristics poses difficulties due to regional and intra-specific variations [18].

The rapid development of molecular biology, the application of the mitochondrial genome, and the partial sequences of COI (cytochrome c oxidase subunit I), 16S rRNA, 12S rRNA, and calmodulin genes have all enabled a greater understanding of the diversity of gastropods at various levels, in terms of population, varieties, species, and so on [19,20,21,22,23,24,25]. Until recently, efforts on molecular taxonomy have been focused on the higher taxonomic categories above the species level. A phylogenetic tree of 72–138 *Conus* species with known diets was obtained on the basis of mitochondrial 16S rRNA and nuclear calmodulin gene sequences, and a distinctive clustering of species with similar diets was observed [26,27]. More details on a phylogenetic basis were established [28] to evaluate morphological criteria and characterize the genetic discontinuity so that *Conus* members can be identified based on gene sequence data from 16S rRNA, COI, and a four-loop conotoxin gene. Monophyly of the Conoidea, characterized by a venom apparatus, has not been questioned; however, subdivisions within the Conoidea and the relationships among them are controversial, mostly because of the uncertainty around the extensive morphological and anatomical variations [29,30]. In summary, a molecular perspective can aid in the phylogenetic classification of Conoidea. Hence, phylogenetic analyses are still essential in determining patterns of speciation and divergence.

### 2.2. Diverse Conotoxins and Targets

Predatory cone snails have long been of interest because of their highly evolved hunting strategies that employ conotoxins to paralyze prey [2]. Cone snails move slowly in an environment of fast-moving prey, which presents a major survival challenge to these predators. However, they have overcome this problem by developing a highly sophisticated venomous apparatus, which is responsible for the synthesis, storage, and delivery of a huge diversity of conotoxins [31].

**Figure 2:**
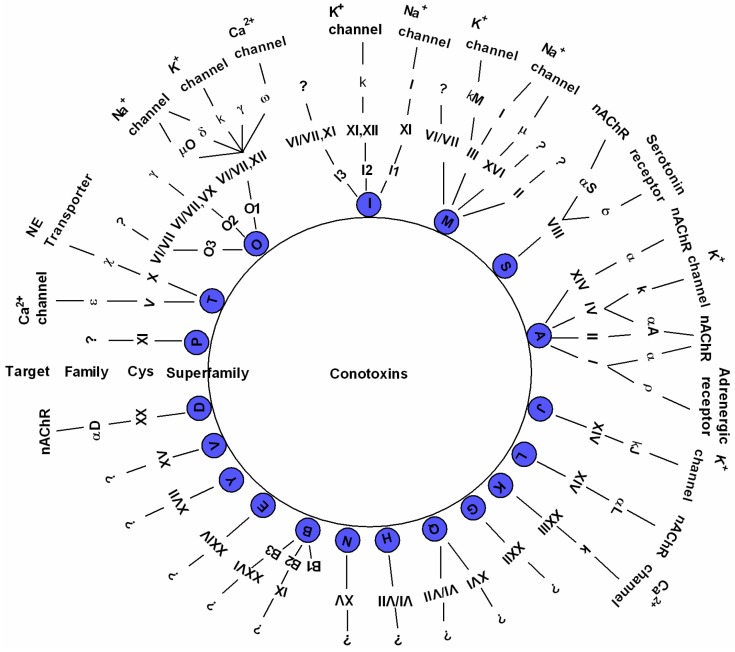
Classification of conotoxins (modified from [34,35]). On the basis of their conserved signal sequence homology, framework, and target receptor, conotoxins are classified into various superfamilies and families. NE: norepinephrine; nAChR: nicotinic acetylcholine receptor.

About 1800 mature conotoxin sequences are available to date, and this number is increasing rapidly as the costs of transcriptome and proteome sequencing continue to reduce [7,8,32]. These diverse conotoxins were originally organized into various superfamilies with the help of two sequence elements, namely the conserved signal sequence and the characteristic cysteine framework. Currently, conotoxins can be classified into 26 gene superfamilies (A, B1, B2, B3, C, D, E, F, G, H, I1, I2, I3, J, K, L, M, N, O1, O2, O3, P, S, T, V, and Y) [33]. Each superfamily can be further divided into several families according to the array of cysteine frameworks. For example, the A-superfamily conotoxins include four cysteine frameworks (I, II, IV, and XIV) and are categorized into α, αA, and κA families; the M-superfamily includes five cysteine frameworks (II, XIV, III, VI, and VII), and is separated into µ and ψ families; the O-superfamily is composed of four cysteine frameworks (XII, XV, VI, and VII), and is classified into δ, µ, O, ω, κ, and γ families [11,14]. A schematic illustration is drawn in Figure 2 to summarize the updated 19 major gene superfamilies, frameworks, families, and ion channel-target networks identified to date [34,35].

The molecular variety of conotoxins mirrors the diversity of their molecular targets [11,14,15,16]. Due to their high specificity and affinity to ion channels, various conotoxins can also be categorized into nicotinic acetylcholine receptor conotoxins (nAChR-conotoxins), sodium channel-targeted conotoxins (Na^+^-conotoxins), potassium channel-targeted conotoxins (K^+^-conotoxins), and calcium channel-targeted conotoxins (Ca^2+^-conotoxins) [11]. Among them, α-, μ-, and ω-conotoxins are the most characterized families so far. Not only is the spectrum of the molecular targets expanding, but the diversity of different sites in a given molecular target also continues to surprise researchers.

### 2.3. Different Distribution and Ecology

**Figure 3:**
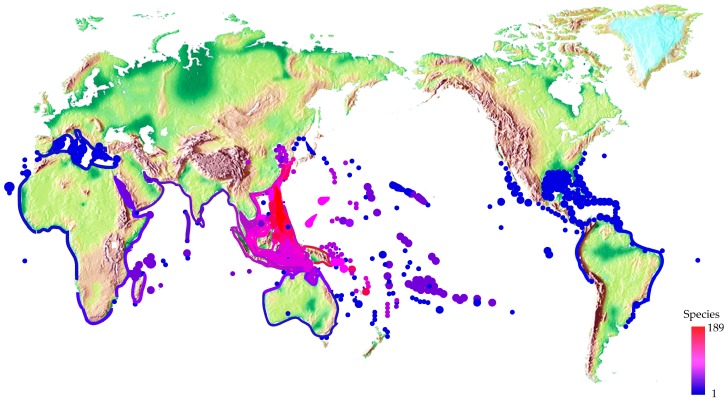
Worldwide distribution of cone snails. Spot colors stand for various species number.

Cone snails are the most diverse genus of marine invertebrates and contribute substantially to the great biodiversity in the tropical Indo-Pacific reef environments [36]. Most cone snails are widely distributed throughout all tropical oceans comprising a quarter of the earth’s ocean area, yet more than 60% of their habitation occurs in the Indo-Pacific region (Figure 3). A few species have adapted to cooler temperate ocean environments, such as *C. californicus*, which is found on the North American Pacific coast [37].

Over 20 species have been observed to co-occur on certain reef platforms, with a maximum of 27 species in Indonesia [38,39]. In more recent papers, 36 *Conus* species were reported on the reef platform fringing Laing Island and 32 species on the four small reefs near Madang of the Northeast Papua Guinea [27]. *C. anemone* and *C. victoriae* are the dominant species in intertidal habitats along the inner region of the Dampier Archipelago, and both reside predominantly under rocks, on sand or limestone substrates [40]. Inter- and sub-tidal regions of the Indian coasts contain nearly 100 *Conus* species, but 16 of the reported species are still currently placed on the list of unverified species due to a lack of sufficient information [41]. However, some considered as unverified species, such as *C. generalis* and *C. litoglyphus*, have been confirmed as a species native to Indian Coastal waters [27,40]. China’s coastal waters have more than 60 species, mainly distributed in the Xisha Islands, Hainan Island, Taiwan Island, and other tropical areas, and the vermivorous *C. betulinus* is the dominant *Conus* species inhabiting the South China Sea [7].

## 3. Multi-Omics Sequencing for High-Throughput Identification of New Conotoxins

Cone snails aroused the interest of some biochemists in the mid-20th century, because of the numerous cases of human injury; several fatalities were recorded due to stings inflicted by these snails [42,43]. After subsequent investigations, a correlation was established between the toxicity of their venoms to vertebrates and their prey type [44]. The first conotoxin was isolated from the venom of the piscivorous *C. geographus* in 1978 [45]. To date, more than 100 natural conotoxin peptides have been purified from the crude venom of cone snails via multi-step chromatography [14].

Cone snails are precious biological resources for marine medicine acquisition. However, researchers face incompatibility problems in the collection and execution of living individuals of the endangered *Conus* species. Moreover, there are many obvious disadvantages to extracting and purifying conotoxins, such as its time-consuming, laborious, high-cost, and low-yield nature, and it could be a substantial waste of bioresources and even cause serious ecological damage.

PCR technology was invented in 1983 [46], with the first conotoxin gene obtained in 1992 [47]; subsequently, PCR has become an important clue for screening novel conotoxin genes. With primes designed from the conservative sequence of each superfamily, PCR amplification has been employed to deal with genomic DNAs, cDNAs, or cDNA libraries in subsequent decades [48,49,50].

**Figure 4:**
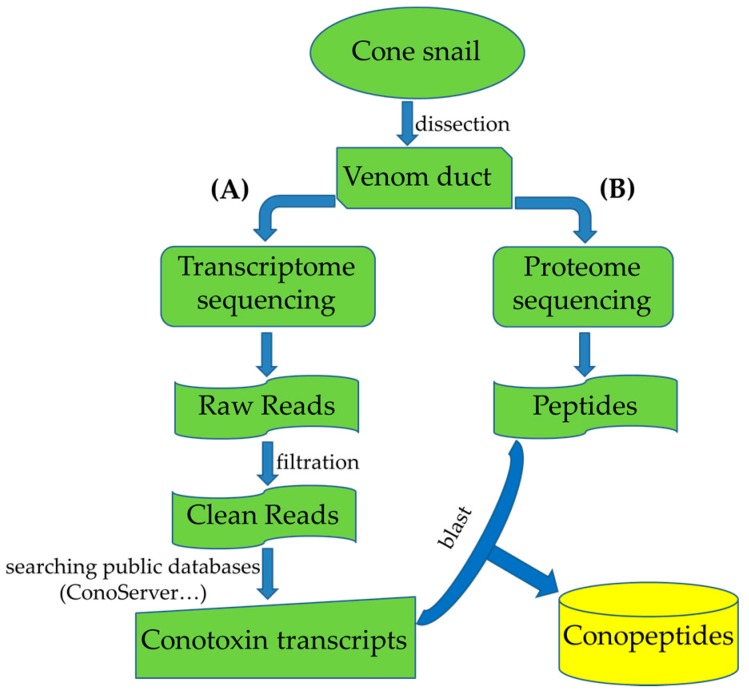
High-throughput identification of new conotoxin transcripts (**A**) and conopeptides (**B**) by transcriptome and proteome sequencing, respectively. More details about sequencing and data analysis can be found in several recent papers [7,9].

In recent years, transcriptomics has developed rapidly with the application of next-generation sequencing technology (Figure 4A) in a cost-effective manner on account of its high throughput sequencing and massive bioinformation analysis capacity [51]. A large number of new conotoxin genes from different species were obtained quickly and efficiently when this technology was used in studies of transcriptomes of the *Conus* venom duct [7]. Transcriptome analysis can provide a comprehensive understanding of mRNA information, including almost all types of the mRNA and their transcription quantity data, from tissue(s) or cell(s) at specific developmental stages or functional status, and can provide a comprehensive reflection of gene transcription within a dynamic scope.

The first report of a *Conus* transcriptome in 2011 was achieved by a team at the University of Utah, which was led by Dr. Olivera BM, a pioneer in the study of conotoxins [52]. The study was the first to show that conotoxins are highly expressed within the venom duct of *Conus* species (*C. bullatus*), and described the first bioinformatics pipeline for high-throughput discovery and characterization of conotoxins. The study also identified 30 putative conotoxin sequences.

From 2013 to 2016, Professor Alewood PF and his team at the University of Queensland completed multi-omics studies of six different *Conus* species, which have improved our understanding of the diversity of conotoxins [1,2,9,32,53,54,55]. Their most representative research in 2013 employed an integrated approach, combining the next-generation transcriptome sequencing with high sensitivity proteomics (Figure 4), to investigate how *Conus* can generate impressive diversity of conotoxins, and a total of 105 conopeptide precursor sequences from 13 gene superfamilies (including five novel superfamilies) were identified from the venom duct of *C. marmoreus* [9]. Interestingly, for the first time, they observed that an average of 20 conopeptides were generated from each conotoxin precursor through a procedure of variable peptide processing. Hence, they estimated that over 2000 conotoxins could be generated in the venom by a single *C. marmoreus* specimen, given that 105 conotoxin precursors were identified from the transcriptome sequencing.

Soon afterwards, Alewood’s team performed an analysis of venom duct transcriptome of *C. marmoreus* with the application of a new algorithm, ConoSorter, and they identified 158 novel conotoxin transcripts and another 13 novel conotoxin gene superfamilies. However, only 106 of these 158 transcripts were confirmed by peptide mass spectrometry, indicating that the effectiveness of ConoSorter is still necessary to be proved [53].

Over the past three years, the algorithm ConoSorter has been improved continuously and has been applied to identify conotoxin precursors from the transcriptome data of five different *Conus* species. From the transcriptomic data of *C. miles*, ConoSorter retrieved 662 putative conotoxin encoded sequences, comprising 48 conotoxin sequences validated at both transcript and peptide levels [32]. In 2015, the team presented a study of transcriptomes and proteomes of the radular sac, salivary gland, and venom duct of a single *C. episcopatus* specimen and discovered 3305 novel toxin sequences from a single *Conus* specimen—the highest number of conotoxins ever found in one specimen [54]. In another work, by the same team, on the *C. catus* transcriptome, 557 putative conotoxin sequences were identified using the 454 sequencing via programs ConoSorter, SignalP, and ConoServer, but only 104 precursors were ultimately recovered because the majority were rare isoforms and excluded from further analysis [2]. Similar sequencing and analysis strategies were applied in subsequent studies of venom duct transcriptomes of *C. planorbis* [55] and *C. vexillum* [1], and a final list of 182 and 220 transcripts, respectively, were obtained.

To explore as many novel conotoxins as possible, we employed an integrated approach to combine next-generation transcriptome sequencing with traditional Sanger sequencing and established a set of efficient methods for the high-throughput identification and validation of novel conotoxins from cone snails [7]. Based on the transcriptomes of the venom duct and venom bulb of vermivorous *C. betulinus*, a dominant *Conus* species inhabiting the South China Sea, we identified a total of 215 conotoxin transcripts within 38 superfamilies or groups, of which nine superfamilies were reported for the first time. We also performed a transcriptomic survey of ion-channel-based conotoxins [56] for the development of conotoxins as potential drugs to treat ion-channel related human diseases. Interestingly, more than 20 conotoxins with potential insecticidal activity were screened out [57] from our transcriptome-based dataset [7] by a homologous search with a reported positive control (IMI from *C. imperialis*) as the query. Two of them were further validated as presenting high insecticidal activity [57], which supports their further study in field investigations as a potential insecticide.

Studies of conotoxins using multi-omics methods have been developing rapidly in recent years. Seventeen articles about venom gland transcriptome research of cone snails have been published to date [1,2,7,8,9,10,32,52,53,54,55,58,59,60,61,62,63]. The related data of 67 transcriptomes, involving a total of 30 *Conus* species, are available from the NCBI. Massive new genes and their encoded toxin peptides can be discovered from this treasure house of marine drugs.

## 4. Structural Prediction with Protein Structure Fingerprinting for Novel Conformations

Over the last few decades, conotoxins have been an important subject of pharmacological interest. As we know, the biological activities of conotoxins are affected by their sequences as well as their structural conformations. A knowledge of conformation is critical to exploiting drug development for conotoxins. As conotoxin peptides usually consist of 10–30 amino acid residues, the conformations are mainly determined by nuclear magnetic resonance (NMR) spectroscopy, X-ray crystallography, or computational prediction approaches. However, except for the more complex procedures, these approaches offer limited conformations under certain circumstances. To date, a large amount of conotoxin data has been collected and compiled in the ConoServer database [64,65]. About 160 conotoxins with determined 3D structures are now available in the Protein Data Bank (PDB); however, for over 8000 conotoxins, only sequence information has been recorded, and 3D conformations have not yet been obtained. Therefore, the challenge is determining how this information can be collected.

One innovative approach—protein folding shape codes (PFSCs) [66], which we established—is able to provide comprehensive conformations for conotoxin peptides. A set of 27 PFSCs completely covers the folding shapes of five successive amino acids, simply represented by the 26 alphabet letters and the “$” symbol as a digitized expression. Consequently, any conformation can be expressed by a string in alphabetical letters comprising a protein structure fingerprint, which can be aligned to directly display the similarity or dissimilarity of conotoxins. For example, the letter A represents a typical alpha-helical fold, and the letters H, D, V, L, Y, and P are for folds with partial alpha-helical similarity. The letter B represents a typical beta-strand fold, and E, G, V, J, M, and S are for folds with partial beta-strand similarity. C, F, L O, $, N, Q, R, I, T, K, X, U, Z, and W mostly relate to irregular folds of the elements of the tertiary structure fragment. The beauty of this approach is that a set of 27 PFSCs covers the complete folding space for the five amino acid residues. Meanwhile, each PFSC vector can be transformed from each other by a skeleton relationship according to the partial sharing of folding similarity.

Here, examples of the conformations of three different conotoxins are described using protein structure fingerprinting with the 27 PFSCs. These examples demonstrate how the conformations are presented for a given 3D structure, and how the predicted conformations are obtained from the sequences. The first structure is conotoxin pl14a with 25 amino acid residues (left column in Table 1). Isolated from vermivorous cone snails, it has potent activity in both nAChR and a voltage-gated potassium channel subtypes [67]. Its structure with 20 conformations is available in PDB (ID: 2FQC), which was determined by NMR spectroscopy. The second structure is alpha-Conotoxin EI with 25 amino acid residues (middle column), originally purified from the venom of *C. ermineus*. Alpha-Conotoxin EI targets neuronal nicotinic acetylcholine receptors but antagonizes neuromuscular receptors [68]. Its structure with 13 conformations is available in PDB (ID: 1K64), which was determined by NMR spectroscopy. The third structure is Omega-conotoxin MVIIA with 26 amino acid residues (right column). It has a range of selectivity for different subtypes of the voltage-sensitive calcium channel [69]. Its structure with 17 conformations is available in PDB (ID: 1K64), which was also determined by NMR spectroscopy. The detailed conformation images, the conformation descriptions for given 3D structures, as well as the conformation predictions from the sequences for these three structures are summarized in Table 1.

The conformations with a given structure can be well described. Section A in Table 1 displays the protein structure fingerprints of 20 conformations of conotoxin pl14a, 13 conformations of Alpha-conotoxin EI, and 17 conformations of Omega-conotoxins MVIIA. Usually, the folding changes are difficult to observe in related structural images; however, the fine differentiations of folding shapes between multiple isomers are easily revealed using a protein structure fingerprint consisting of the 27 PFSCs.

The NMR spectroscopy approach, indeed, measures the nature of conotoxin conformations in fluctuation. Furthermore, with protein structure fingerprinting, the conformation alignment of the isomers for each structure reveals the locations of folding fluctuations or stability along sequences. In the 2FQC structure, it is apparent that in 20 isomers most of the local folds are alpha helices alike, and more folding changes happen in the N-terminus while stable conformations appear in the C-terminus. In the 1K64 structure, a fragment (10–13) of the 13 isomers has a stable conformation, but other parts showed more fluctuation in conformations. In the 1CNN structure, a fragment (7, 9, 13–19) of the 17 isomers has a stable conformation, but other parts showed more changeable conformations. Together, 2FQC has relatively longer alpha helices in conformation; 1CNN has a stable fragment with alike alpha helices; 1K64 has many folding variations with shorter fragments.

The comprehensive folding variations in each conformation can be predicted directly according to the sequence. Section B at the bottom of Table 1 displays the complete variations of local folding shapes for three structures, which are actually the ensembles of folding shapes for five successive amino acids along the conotoxin sequences. In fact, the complete variations provide rich information to cover all possible changes in local folding shapes. It is noted that the possible types and numbers of folding shapes are altered for each of the five successive amino acids along the conotoxin sequence. For example, the predicted folding variations for conotoxin pl14a indicate stability in the C-terminus. However, the fragment with the sequence “RAGIG” (12–16) has the highest number of folding shape variations, indicating the location with the most flexibility of folding changes in the conotoxin pl14a. The predicted folding variations in Section B of Table 1 are significant, suggesting which locations have a more flexible selection in the folds and less selection for local folds. Meanwhile, all folding changes in the isomers for given 3D structures are encompassed by the complete folding variations found from prediction. Although each structure in Section A of Table 1 has different conformations with altered folds, all these folds are totally covered by the folding variations in Section B and are marked in yellow. In other words, any conformation of isomer for each conotoxin structure in Section A of Table 1 can be well predicted using the comprehensive folding variations in Section B.

The binding sites for conotoxins in any protein receptor can be described by its protein structure fingerprint with the 27 PFSCs. For example, alpha-conotoxin is a peptide antagonist of nAChRs that has been used as a pharmacological probe and investigated as a drug lead for nAChR-related disorders [70]. The co-crystal structure (PDB ID: 5T90) is an alpha-conotoxin binding to human α3β4 nAChR. A detailed image of the structure and its binding sites using protein structure fingerprinting is presented in Table 2. In this structure, the alpha-conotoxin is defined as Chain F (yellow color), which is surrounded by Chain A and Chain C of nAChR (images on the left side of Table 2). The binding sites were determined by an 8 Å distance of interaction of all atoms from alpha-conotoxin. Hence, it was revealed that the binding site is formed by five fragments from Chains A and C. It is hard to study the conformation of binding sites with an image or computational molecule modeling approach. However, the topological space of binding sites can be explicitly described using PFSCs. The five fragments with sequences and folding descriptions are displayed in the bottom section of Table 2, which may better illustrate how alpha-conotoxin peptides act as antagonist leads for nAChR-related disorders. Furthermore, to query the similar fingerprint of the known binding sites with other proteins, multiple protein targets may be discovered with high-throughput screening of protein databases. This approach may be better for understanding various interactions between conotoxins and protein receptors, such as nAChR in nerves and muscles [71], voltage-dependent sodium channels [72], potassium channels [73], sodium channels in muscles [74], and N-type voltage-dependent calcium channels [75].

In summary, the protein structure fingerprint with 27 PFSC vectors is able to provide rich information for studying conotoxin conformations. First, based on a given 3D structure, the protein structure fingerprint is able to provide a complete description of conotoxin conformations. Second, based on sequences, the protein structure fingerprint can provide comprehensive folding variations to predict the conotoxin conformations of unknown 3D structures. Third, the protein structure fingerprint may provide another means of exploring the mechanisms of interactions between various conotoxins and related protein targets.

## 5. Recent Advances in Conotoxins for Drug Development

The therapeutic potential of conotoxins is ascribed to their special ion-channel targets in nervous systems [76]. Thus, conotoxins have potentially wide applications in the fields of neuroscience research and development. Many conotoxins are proving to be valuable as research tools, drug leads, and drugs [14,15,77]. We performed a detailed survey of US patent literature covering conotoxins to determine their potential therapeutic applications.

**Figure 5:**
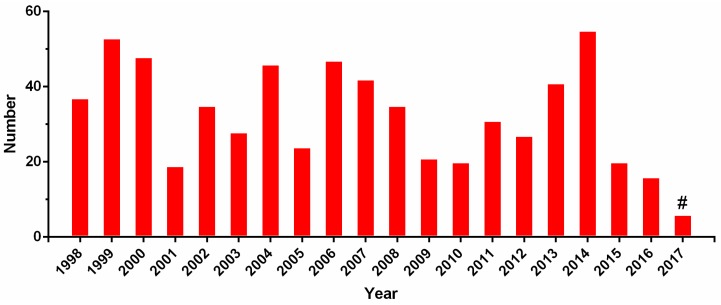
The number of patents for conotoxins per year. Note that # indicates the incomplete number counted to 2017.

**Figure 6:**
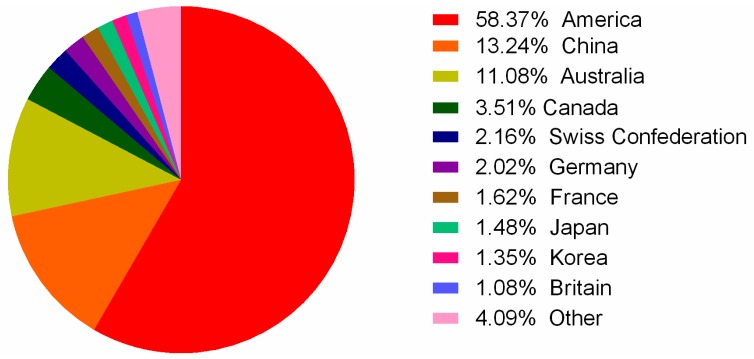
The national distribution of patents for conotoxins.

The State Intellectual Property Office of China (SIPO; [78]) was used as the primary search, while the United States Patent and Trademark Office (USPTO; [79]) Patent Office for Europe (EPO; [80]) and World Intellectual Property Organization (WIPO; [81]) assisted the search. All these databases were searched with the following keywords: conotoxin, conopeptide, conantokin, contryphan, and contulakin. The search period was set from 1998 to 2017. From this search, 811 patents, of which 243 were authorized, were obtained. These patents were classified into different families based on continuity relationships. Among these patent families, the majority (451) refer to conotoxin compositions of matter and the remaining 360 primarily cover processes and methods for their applications [82]. We observed that the number of patent applications has fluctuated in the past 20 years, and the maximum number occurred in 2014 (Figure 5). Patent applicants are mainly distributed in the United States, China, and Australia (Figure 6).

To date, several conotoxins have already demonstrated potential therapeutic effects in preclinical or clinical trials (Table 3). The most well-known commercial conotoxin is ω-MVIIA (ziconotide), which has been approved by the America FDA to treat intractable chronic pain in cancer and AIDS patients [12,13,83]. Its introduction into the market not only demonstrated the therapeutic potential of conotoxins but also stimulated more interest from biotechnology companies to support conotoxin research. Those conotoxins currently in clinical trials include an analog of the conotoxin χ-MrIA, which noncompetitively inhibits noradrena-line transporter and is undergoing phase II clinical trials as a treatment for neuropathic pain [84]. Other ω-conotoxins in the clinical trial pipeline include ω-CVID, which successfully completed preclinical studies, but high cytotoxic effects were observed during phase IIa trials [85]. In addition, contulakin-G and conantokin G are, respectively, the specific antagonist against the Neurotensin receptor and NR2B subunit of the NMDA receptor, and are currently in human clinical trials for pain and intractable epilepsy [86,87]. Therefore, an increasing number of conopeptides are undergoing development for the treatment of pathologies including pain, cancer, cardiac infarction, hypertension, Parkinson’s disease, Alzheimer’s disease, epilepsy, and various neurological diseases [3,6,11,14,15]. Several conopeptides (Table 3) reaching human clinical trials have already established the *Conus* pharmacopoeia as a rich source of therapeutics for neurological disorders [14].

## 6. Conclusive Remarks

The diversity of cone snails offers a promising prospect for drug discovery, with the rapid development of genomic/proteomic data and bioinformatics methods. However, despite the great advances of conotoxins in drug development, the incapability of these conotoxins to cross the blood–brain barrier results in their dependence on intrathecal administration. This remains a major challenge in the therapeutic application of conotoxins. Elevation of in vivo stability and efficient absorption, as well as 3D structural modifications as described above, will also greatly boost their clinical success.

## Figures and Tables

**Table 1:** Conformation descriptions of three conotoxin examples.

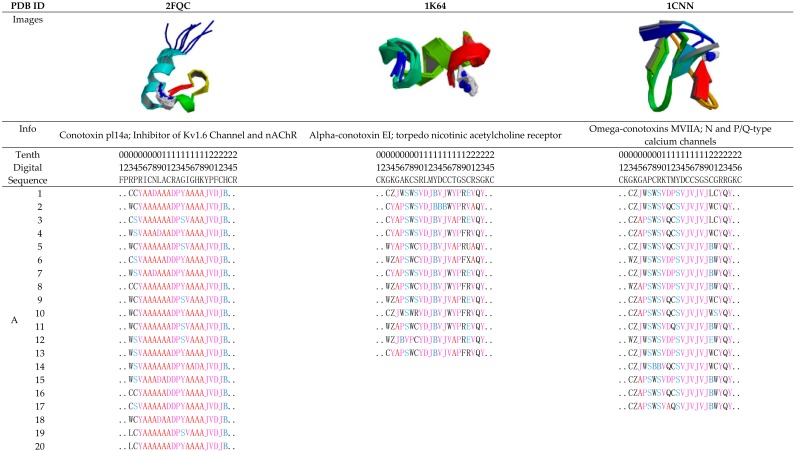 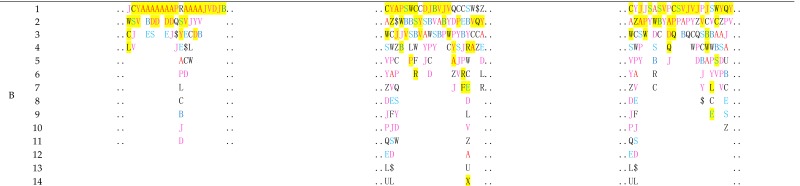

Notes: The first row is the structure entity (PDB ID). The following rows, respectively, are conformation images presented by a solid ribbon format, conotoxin information, rules of position, and amino acid sequence. Section A lists the conformation descriptions of the structure fingerprint by PFSCs according to the given 3D structures. Section B displays the predicted folding variations according to the conotoxin sequences, which are ensembles of folding shapes for five successive amino acids. The PFSC folding shapes are marked by different colors: red is for a typical helix fold; blue is for a typical beta fold; pink and light blue are for folds with a partial helix or beta; black is for irregular folds.

**Table 2:** Binding site description using a protein structure fingerprint for an alpha-conotoxin (LsIA) binding with human α3β4 nicotinic acetylcholine receptor.

**Alpha-Conotoxin in Structure (PDB ID: 5T90 for LsIA)**				
Chain	Start	End	Sequence	PFSC
F	1	17	SGCCSNPACRVNNPNIC	**..AAAJVAAAAAJVA..**
Image of LsIA (yellow; PDB ID: 5T90) binding with human α3β4 nicotinic acetylcholine receptor			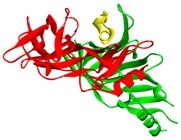	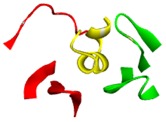
			Chain A (red); Chain C (green); Conotoxin as Chain F (yellow)	Conotoxin binding site with five fragments
**Binding Fragments of nAChR for Alpha-Conotoxin (PDB ID = 5T90)**				
Chain	Start	End	Sequence	PFSC
A	53	57	WQQTT	**RBBEE**
A	102	116	LARVVSDGEVLYMPS	**BLREWYQSBBEBEWR**
A	155	165	TTENSDDSEYF	**CYJVAJVAAAP**
C	141	149	GSWTHHSRE	**WSVAJWZAD**
C	183	193	VTYSCCPEAYE	**BEWYAJVPREE**

Notes: The images are presented in a solid ribbon format. The image on the left is colored to distinguish each chain; the image on the right presents the binding site formed by five fragments. The binding fragments were determined by the 8 Å distance of interaction of all atoms from the alpha-conotoxin. The PFSC folding shapes are marked as various colors: red is for a typical helix fold; blue is for a typical beta fold; pink and light blue are for folds with a partial helix or beta; black is for irregular folds.

**Table 3:** Therapeutic applications of conotoxins [14,15,77].

Clinical Application	Conopeptide	Molecular Target	Clinical Status	Reference
Pain	ω-MVIIA (Ziconitide)	Ca^2+^ channel (CaV2.2) N-type calcium channels/blocker	FDA-approved	[83]
Pain	χ-MrIA (Xen2174)	Norepinephrine transporter	Phase IIa *	[84]
Pain	ω-CVID (AM336)	Ca^2+^ channel (CaV2.2) N-type calcium channels/blocker	Phase IIa *	[85]
Pain	Contulakin-G (CGX-1160)	Neurotensin receptor	Phase Ib *	[86]
Pain/Neuro protection	Conantokin-G (CGX-1007)	NMDA receptor (NR2B)	Preclinical *	[87]
Pain	α-Vc1.1 (ACV1)	nAChR (α9α10)	Phase II *	[88]
Myocardial infarction	κ-PVIIA (CGX-1051)	K^+^ channel (K_V_1)	Preclinical	[89]
Neuropathic pain	μO-MrVIB (CGX-1002)	Sodium channels/subtype selective blocker	Preclinical *	[90]

Note: * indicates that development of these conotoxins has been terminated.
